# Response of cyanobacteria and phytoplankton abundance to warming, extreme rainfall events and nutrient enrichment

**DOI:** 10.1111/gcb.14701

**Published:** 2019-07-04

**Authors:** Jessica Richardson, Heidrun Feuchtmayr, Claire Miller, Peter D. Hunter, Stephen C. Maberly, Laurence Carvalho

**Affiliations:** ^1^ Centre for Ecology & Hydrology Lancaster Environment Centre Lancaster UK; ^2^ Biological and Environmental Sciences, Faculty of Natural Sciences University of Stirling Stirling UK; ^3^ School of Mathematics and Statistics University of Glasgow Glasgow UK; ^4^ Centre for Ecology & Hydrology Penicuik UK

**Keywords:** climate change, experiment, harmful algal bloom, lake, mesocosm, *Microcystis*, multiple stressors

## Abstract

Cyanobacterial blooms are an increasing threat to water quality and global water security caused by the nutrient enrichment of freshwaters. There is also a broad consensus that blooms are increasing with global warming, but the impacts of other concomitant environmental changes, such as an increase in extreme rainfall events, may affect this response. One of the potential effects of high rainfall events on phytoplankton communities is greater loss of biomass through hydraulic flushing. Here we used a shallow lake mesocosm experiment to test the combined effects of: warming (ambient vs. +4°C increase), high rainfall (flushing) events (no events vs. seasonal events) and nutrient loading (eutrophic vs. hypertrophic) on total phytoplankton chlorophyll‐*a* and cyanobacterial abundance and composition. Our hypotheses were that: (a) total phytoplankton and cyanobacterial abundance would be higher in heated mesocosms; (b) the stimulatory effects of warming on cyanobacterial abundance would be enhanced in higher nutrient mesocosms, resulting in a synergistic interaction; (c) the recovery of biomass from flushing induced losses would be quicker in heated and nutrient‐enriched treatments, and during the growing season. The results supported the first and, in part, the third hypotheses: total phytoplankton and cyanobacterial abundance increased in heated mesocosms with an increase in common bloom‐forming taxa—*Microcystis* spp. and *Dolichospermum* spp. Recovery from flushing was slowest in the winter, but unaffected by warming or higher nutrient loading. Contrary to the second hypothesis, an antagonistic interaction between warming and nutrient enrichment was detected for both cyanobacteria and chlorophyll‐*a* demonstrating that ecological surprises can occur, dependent on the environmental context. While this study highlights the clear need to mitigate against global warming, oversimplification of global change effects on cyanobacteria should be avoided; stressor gradients and seasonal effects should be considered as important factors shaping the response.

## Graphical abstract

Cyanobacteria are expected to benefit from a warmer climate, especially in nutrient‐rich waters. However, other important climate change factors—more extreme rainfall events—could affect this response (e.g. loss through flushing). This mesocosm study tested the combined effects of warming, extreme rainfall events and nutrient loading on cyanobacterial abundance. Warming increased the abundance of bloom‐forming taxa, but in combination with very high nutrient loading resulted in a negative, not positive, interaction. The impact of extreme rainfall events was only apparent in the winter. Stressor gradients and season should be considered as important factors shaping the response to global change.

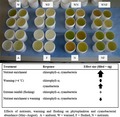

## 1 INTRODUCTION

Blooms of cyanobacteria are a major threat to freshwater quality and global water security (Codd, Morrison, & Metcalf, 2005; Steffensen, 2008), driven primarily by the anthropogenic over‐enrichment of freshwaters (Taranu et al., 2015). However, there is a broad consensus that elevated water temperatures also promote the proliferation of cyanobacterial blooms (Paerl & Huisman, 2008; Richardson et al., 2018). This is because cyanobacteria have a number of traits which may provide them with an advantage in warmer conditions (Carey, Ibelings, Hoffmann, Hamilton, & Brookes, 2012; Mantzouki, Visser, Bormans, & Ibelings, 2016). For example, many bloom‐forming cyanobacteria reach their maximum growth rate at higher temperatures than other phytoplankton (Butterwick, Heaney, & Talling, 2005; De Senerpont Domis, Mooij, & Huisman, 2007; Reynolds, 2006), and benefit from warming‐enhanced internal cycling of nutrients (McKee et al., 2003) and greater water column stability (Huber, Wagner, Gerten, & Adrian, 2012; Jöhnk et al., 2008; also see Carey et al., 2012). Studies over a range of scales—experimental (Lürling, Oosterhout, & Faassen, 2017), single water body (Taranu, Zurawell, Pick, & Gregory‐Eaves, 2012; Zhang, Duan, Shi, Yu, & Kong, 2012) and regional (Beaulieu, Pick, & Gregory‐Eaves, 2013)—provide ample evidence that higher temperatures promote higher cyanobacterial abundance and thus severely affect our ability to control blooms (Havens & Paerl, 2015). The threat of cyanobacterial blooms is, therefore, expected to increase in response to rapid global warming.

The response of cyanobacteria to warming and nutrient enrichment may, however, be complicated by other large‐scale environmental changes which can alter phytoplankton growth and community structure. This includes the predicted increase in extreme stormy weather (IPCC, 2013). More extreme rainfall events are now being observed globally (Lehmann, Coumou, & Frieler, 2015) and, in particular, are predicted to increase during the summer months at mid‐ to high latitudes (Christensen & Christensen, 2003). These perturbations can strongly affect phytoplankton abundance and communities; directly, through loss of biomass to the outflow (Reynolds, Maberly, Parker, & Ville, 2012; Sadro & Melack, 2012) and indirectly, through changes in selection pressures that affect community composition and diversity such as changes in nutrient concentrations, mixed depth and turbidity (James et al., 2008; Padisák, Köhler, & Hoeg, 1999; Sadro & Melack, 2012). Depending on the nature of the event (e.g. timing, frequency and duration), hydrological context (e.g. antecedent weather) and environmental context (e.g. nutrient source, lake morphometry, catchment geology and land use), high rainfall events may have positive or negative impacts on cyanobacteria (Paerl & Huisman, 2008; Reichtwaldt & Ghadouani, 2012). For example, a ‘perfect storm’ of a large pulse of nutrients followed by a dry period of no flushing could benefit cyanobacteria (Paerl et al., 2016) while a ‘turbid‐event’ (Perga, Bruel, Rodriguez, Guénand, & Bouffard, 2018) can have long‐term negative effects because of decreased light availability (James et al., 2008). Nutrient loading in turn depends on the source of nutrients, that is point or diffuse (Elliott, Jones & Page, 2009), catchment geology and antecedent weather (Perga et al., 2018; Reichtwaldt & Ghadouani, 2012). This complexity results in a wide range of environmental change scenarios which can impact phytoplankton dynamics in different ways.

The inherent complexity of hydrologically induced change in lakes, as well as the rarity of extreme events in the real world, makes pulse disturbances hard to systematically study across spatial and temporal scales using natural lakes. Experimentation offers a means to disentangle the different aspects of high flow events which are expected to impact phytoplankton communities in many ways, such as biomass loss, nutrient loading, changes in colour, increases in turbidity and changes in mixed depth. Lake mesocosm studies are especially useful to explore the effects of multiple stressors, by allowing environmental conditions to be manipulated while retaining ecosystem complexity (Fordham, 2015; Stewart et al., 2013). To improve our ability to forecast the effect of global warming on cyanobacteria, we need to take a complete view of future conditions, incorporating ‘event‐focused’ pulse disturbances as well as ‘trend‐focused’ press climate effects (Jentsch, Kreyling, & Beierkuhnlein, 2007; Michalak, 2016). To our knowledge, only one mesocosm study has tested the effects of short‐term high flow events, focusing on the effects of pulses of ‘terregenic’ material from the catchment (Graham & Vinebrooke, 2009). No study has yet explored the effects of multiple stressors, including high flow pulse events, on the phytoplankton community, particularly on potentially harmful blooms of cyanobacteria.

Here we describe a shallow lake mesocosm experiment that tested potential interactions between warming, nutrient enrichment and extreme rainfall events (flushing of biomass) on the abundance and composition of cyanobacteria and total phytoplankton abundance (as measured by chlorophyll‐*a*). The levels of each treatment were chosen to simulate current and future scenarios. Small, shallow lakes are of particular interest as they are numerically dominant globally (Messager, Lehner, Grill, Nedeva, & Schmitt, 2016; Verpoorter, Kutser, Seekell, & Tranvik, 2014), are especially sensitive to changes in air temperature (Butcher, Nover, Johnson, & Clark, 2015), have a higher exposure to nutrient pressures because of their abundance in lowland, impacted landscapes (Nõges, 2009) and a higher sensitivity to extreme rainfall events because of their smaller volume. We hypothesised that: (a) warming would favour the growth of cyanobacteria over other phytoplankton, in particular taxa with higher temperature growth optima such as *Microcystis* spp. and *Dolichospermum* spp.; and (b) that the effect would be synergistic with nutrient addition that is greater than the sum of their individual effects. We also expected that flushing events would result in the loss of phytoplankton and hypothesised (c) that recovery of overall biomass and composition (phytoplankton compared to cyanobacteria) would depend not only on the nutrient and warming treatment but also on the time of the year. Specifically, we hypothesised (d) that recovery would be quickest in nutrient‐enriched and heated mesocosms, but also during the spring and summer when the conditions for growth, and recovery, would be optimal. Furthermore, we hypothesised (e) that cyanobacteria may be more sensitive to flushing (slower to recover) because of slower growth rates compared to other competing phytoplankton taxa.

## 2 MATERIALS AND METHODS

**Figure 1:**
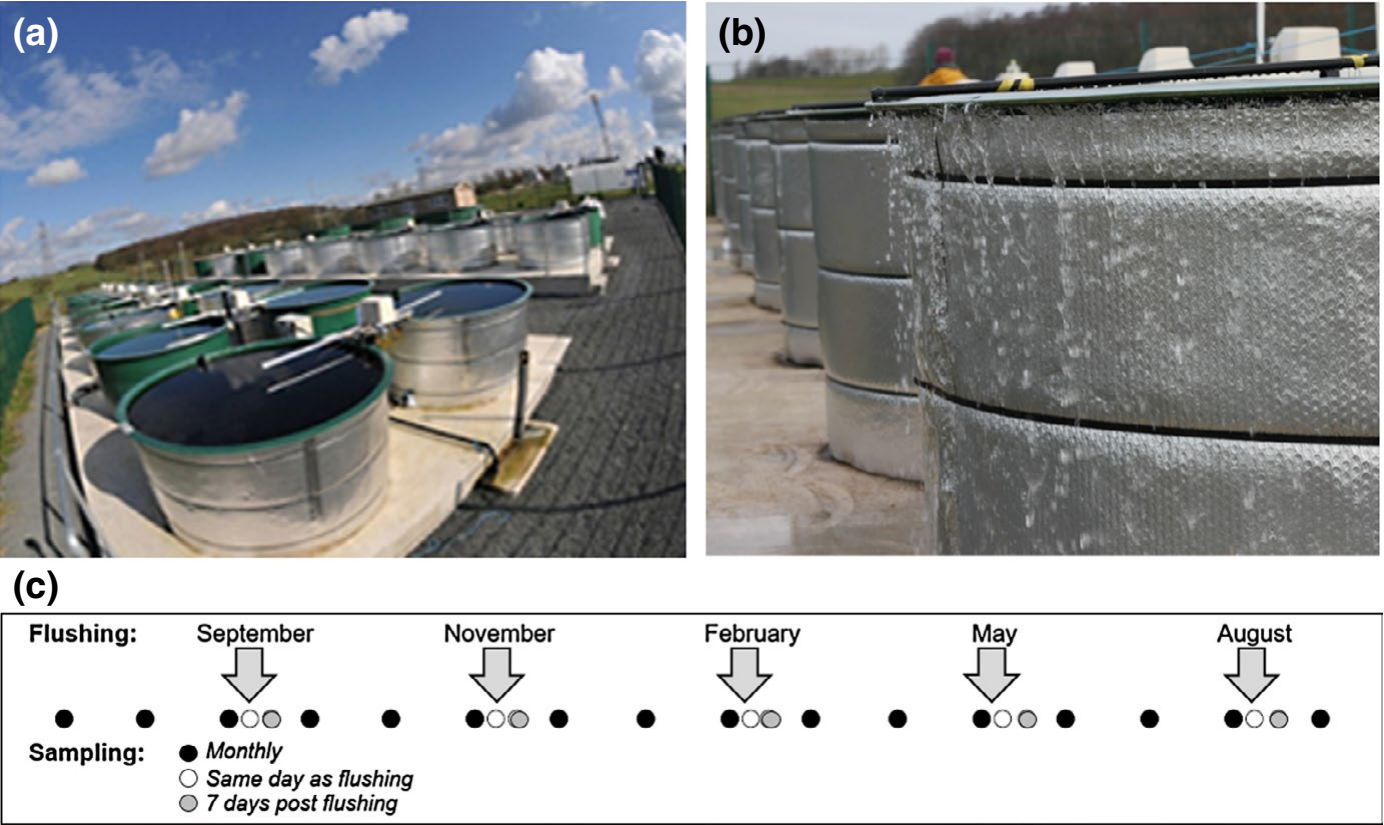
Mesocosm set‐up and experimental design. The mesocosms (3,000 L capacity) are organised into four experimental blocks of eight mesocosms (a); each mesocosm has a mechanical mixing system (white extended arm), a power supply (white box) for the heating system and a heating element which sits above the sediment. Flushing events (arrows) occurred every 12 weeks (c); during each event, 1,500 L of water was pumped into each mesocosm—water was lost by overflowing the top of the mesocosms (b). Sampling occurred every 4 weeks as well as additional sampling which occurred on the day of flushing (post‐flushing), and 1 week after a flushing event before returning to a four‐weekly schedule

A fully factorial experiment combining two temperature treatments, two nutrient treatments and two extreme rainfall treatments was performed in 32 outdoor mesocosms from July 2014 to August 2015 at the Centre for Ecology & Hydrology's Aquatic Mesocosm Facility located in the North West of England (54°1′N, 2°46′W) (https://www.ceh.ac.uk/our-science/research-facility/aquatic-mesocosm-facility). The levels of each treatment were chosen to simulate current and future scenarios; these are described in greater detail in the following sections. The eight treatments (the full cross of each factor) were replicated four times, one replicate randomly assigned to a mesocosm in each experimental block of eight mesocosms (Figure 1; Figure S1).

### 2.1 Description of mesocosms

The mesocosms are free‐standing, open‐topped, non‐transparent and insulated cylinders, measuring 1 m in depth and 2 m in diameter (3,000 L capacity, Figure 1). Each contains a heating element, located 14–15 cm above a 5–6 cm deep mixture of washed sand and lake sediment (in equal proportion), taken from Windermere, a large mesotrophic lake in the English Lake District, UK. Mesocosms were filled with an equal volume of rain water and water from Windermere and were inoculated with phytoplankton, zooplankton and macroinvertebrates, also from Windermere, to simulate realistic natural community compositions (Reynolds & Irish, 2000). Mesocosms were allowed to settle for 13 months, during which macroinvertebrates were restocked twice and also cross‐mixed twice to ensure similar starting conditions. At the start of the experiment, there was no statistically significant difference in chlorophyll‐*a* concentrations between the eight treatments (Table S2); phytoplankton composition data were not available for the start of the experiment. Four adult three‐spined sticklebacks (*Gasterosteus aculeatus*), two of each gender, were sourced from local streams (New Draught and Barton Brook, Lancashire) and were added to each mesocosm. Between capture and inoculation, fish were kept in 30 L glass aquaria, containing untreated water from Blea Tarn Reservoir. Macrophyte populations established from natural seed banks within the sediment. Any water losses from evaporation were monitored and rectified by the addition of deionised water.

### 2.2 Treatments

#### 2.2.1 Warming

Under the worst‐case scenario of ‘business as usual’, it is predicted that global temperatures could rise as much as 4.8°C by the end of the century (IPCC, 2013). In this experiment, half the mesocosm were left at ambient temperatures (unheated mesocosms) to simulate current conditions while the other half were heated to 4°C (heated mesocosms) to simulate future conditions. This is in the upper range of the predicted increase in temperatures under RCP 8.5 predictions (2.6–4.8°C, IPCC, 2013) and complements other mesocosm climate warming experiments (e.g. Feuchtmayr et al., 2009; Feuchtmayr et al., 2019; Urrutia‐Cordera et al., 2017; Yvon‐Durocher et al., 2015).

Water temperature (°C) was recorded every minute by sensors located 40 cm horizontally and vertically (mid‐depth) within each mesocosm and then stored on a data logger. A computer program adjusted the water temperature in heated mesocosms so that it tracked changes in temperature in unheated mesocosms (Figure S2). Temperatures in unheated mesocosms followed a seasonal cycle typical of temperate regions; daily mean temperatures varied between 2.4°C in January and 23.4°C at the end of July. Shallow lakes are often polymictic due to their large surface to volume ratio. While it is recognised that the duration of stratification may increase as a result of climate warming (Wagner & Adrian, 2009), mesocosms contained automatic mixers to prevent thermal stratification (Figure S3). This was so that direct effects of increased water temperature could be assessed. For further details regarding the experimental facility, please see Feuchtmayr et al. (2019).

#### 2.2.2 Nutrient enrichment

In Europe, nutrients are the primary stressor in freshwaters (Nõges et al., 2016), with as many as 72% of shallow lakes having summer nutrient concentrations classified as eutrophic or hypertrophic (data from 452 shallow European lakes, Moe, Schmidt‐Kloiber, Dudley, & Hering, 2013). Despite efforts to reduce nutrient loading, the pressures of agriculture and urbanisation continue to impact lakes. The mesocosms were enriched with nitrogen and phosphorus, half of the mesocosms at high concentrations to create future ‘nutrient‐enriched’ (hypertrophic) conditions and half at lower concentrations to create current ‘ambient nutrient’ (eutrophic) conditions.

A fortnightly load of nitrogen and phosphorus was added to nutrient‐enriched mesocosms to produce final Redfield ratio concentrations (Redfield, 1958) in each mesocosm equivalent to 510 μg/L nitrogen (sodium nitrate) and 70 μg/L phosphorus (trisodium phosphate). Over the course of the experiment, this resulted in average nutrient concentrations of 314 ± 86 μg/L (minimum of 156 µg/L) for total phosphorus (TP) and 1576 ± 298 μg/L (minimum of 745 µg/L) for total nitrogen (TN) which is similar to the upper range of concentrations recorded in natural lakes in agricultural catchments in Europe (Moe et al., 2013). In the 16 ambient nutrient mesocosms, a fortnightly load equivalent to 73 μg/L of nitrogen and 10 μg/L of phosphorus was added. Nutrient analyses from the first few months were higher than expected, indicating that the sediment used was high in nutrients, thus from 17 December nutrient additions were stopped, so that nutrient concentrations did not exceed the desired range for the treatment. This pattern of additions only occurred in ambient nutrient mesocosms as there was no planned upper range for the nutrient‐enriched treatment. Over the course of the experiment, the average TP concentration in the ambient nutrient addition mesocosms was 100 ± 47 μg/L (minimum of 38 µg/L) and the average TN concentration was 692 ± 218 μg/L (minimum of 385 µg/L). Based on average TP concentrations over the duration of the experiment, nutrient‐enriched mesocosms were classified as being hypertrophic while ambient nutrient addition mesocosms were on the eutrophic‐hypertrophic boundary (OECD, 1982).

#### 2.2.3 Extreme rainfall events

Half the mesocosms were exposed to extreme rainfall (flushing) simulations every 12 weeks—five events on: 3 September 2014; 24 November 2014; 17 February 2015; 12 May 2015; and 4 August 2015. While extreme rainfall events are predicted to increase during the summer months at mid‐ to high latitudes (Christensen & Christensen, 2003), this regime was chosen to compare high rainfall events and stressor combinations between seasons. Increased flow to a lake results in many physico‐chemical changes and consequently many potential effects on biological responses. To understand the effects of rainfall events on phytoplankton abundance and composition, these effects should be tested in combination and in isolation. Here, the effect of hydraulic flushing of biomass as a result of increased flow was tested.

**Figure 2:**
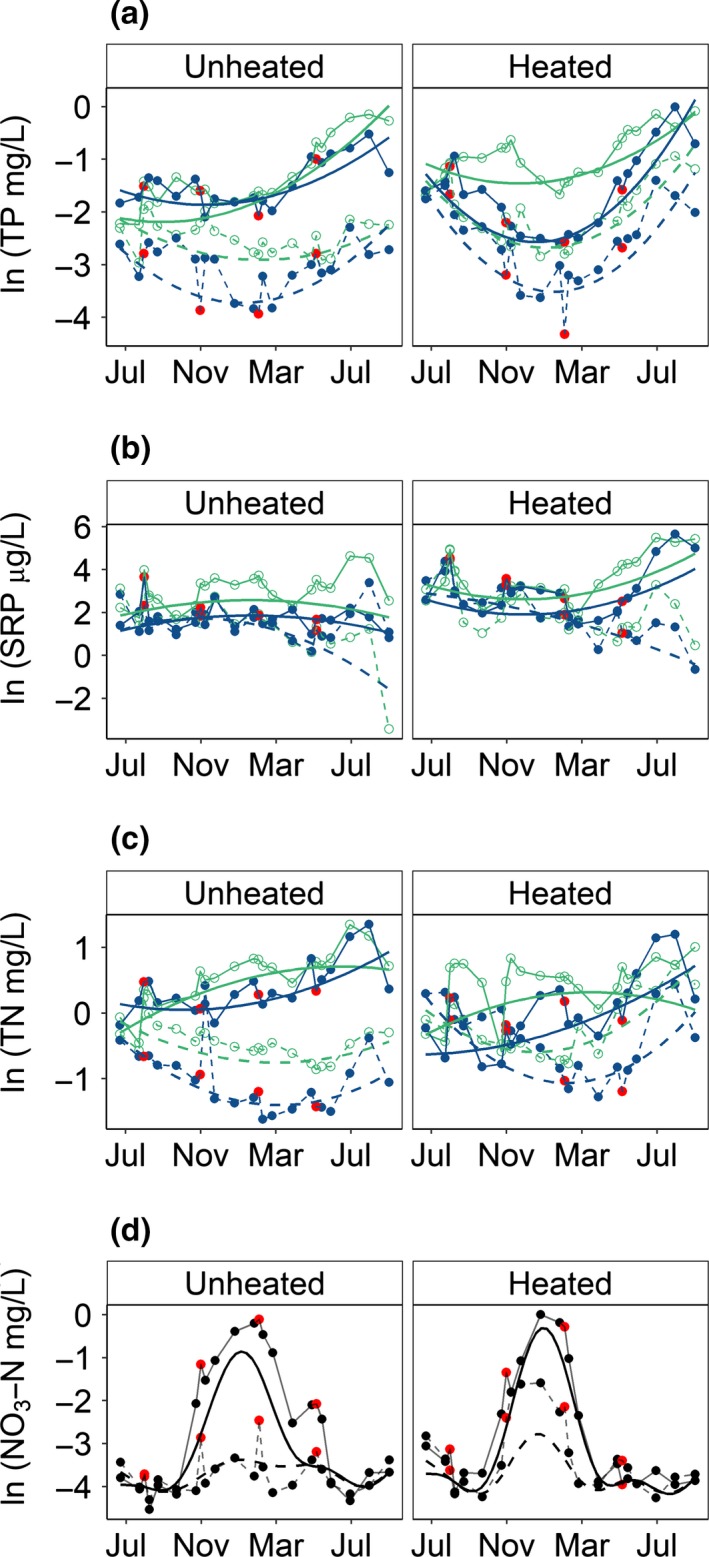
Effect of nutrient enrichment and extreme rainfall events on the concentration of (a) total phosphorus (TP; marginal *R* ^2^ = 0.55), (b) soluble reactive phosphorus (SRP; marginal *R* ^2^ = 0.29), (c) total nitrogen (TN; marginal *R* ^2^ = 0.37) and (d) NO_3_‐N (*R* ^2^ adjusted = 0.59) in ambient and warmed mesocosms over time (July 2014–August 2015). The response of each measured variable is ln transformed (note differences in the original scale); data points are mean responses for the treatment plotted. Smooth lines in panels (a–c) are the predicted fitted responses from the best fitting linear mixed model (Table 1): blue, flushed; green, unflushed; solid line, nutrient enriched; dashed line, ambient‐nutrient—Figure S5 for model confidence intervals. The smooth black lines in panel (d) are predicted fitted responses from the best fitting additive mixed model (Table 2): solid line, nutrient enriched; dashed line, ambient‐nutrient addition. For extreme rainfall treatments (blue lines in panels a–c, all treatments in panel d) red data points show data from the sampling events the day immediately after an extreme rainfall event (nutrients were not sampled on the day after the August flushing event)

During each event, 1,500 L of water (50% of the capacity of a mesocosm) was pumped into each treated mesocosm at a flow rate of 70–100 L/min (duration of 15–21 min), taking care not to disturb the sediment while ensuring homogenous mixing; water was lost by overflowing the top of the mesocosms. Water was sourced from the Blea Tarn Reservoir, Hazelrigg, Lancaster, which was low in nutrients, phytoplankton and total suspended material **(**Table S1). Any dissolved nutrients lost during the flushing event were replaced so that the effects of biomass loss could be isolated from other effects of extreme rainfall events such as increases in nutrient loading and other allochthonous material. This was calculated from the amount of nutrients lost by dilution (see Data S1: Methods for details) and that added by the water used for flushing. Nitrogen was not added to some of the mesocosms as nitrate in the Blea Tarn water exceeded the concentrations within some of the ambient‐nutrient treatments. Despite this, there was no statistically significant effect of flushing on the concentration of soluble reactive phosphorus (SRP) or nitrate (NO_3_‐N; Figure 2; Table 1).

**Table 1:** Summary of ANOVA tables of type III for responses fitted with LMMs. For all measured variables, time is a second order quadratic term in the model

	Chlorophyll‐*a*	TP	SRP	TN
	*F* value	*F* value	*F* value	*F* value
**Time**	**216.5**	**166.5**	**19.3**	**20.8**
**Time** × N	**21.2**	**24.9**	**45.9**	**38.1**
**Time** × W	**5.3**	**12.7**	**13.3**	2.4
**Time** × F	**4.3**	**6.7**	n.a.	**5.9**
**Time** × N × W	**14.9**	2.9	**4.3**	**3.8**
**Time** × N × F	0.1	1.1	n.a.	**4.7**
**Time** × W × F	**5.0**	2.3	n.a.	0.2
**Time** × N × W × F	**10.1**	**8.2**	n.a.	**3.3**
N	**59.8**	**55.8**	**33.8**	**38.6**
W	0.2	3.3	**18.4**	0.0
F	**4.4**	**10.2**	4.0	**7.3**
N × W	**4.9**	1.8	0	**8.6**
N × F	1.7	1.0	**4.3**	0.9
W × F	**7.2**	1.9	n.a.	0.0
N × W × F	3.4	1.4	n.a.	0.4
$R_{\text{marginal}}^{2}$	0.57	0.55	0.29	0.37
$R_{\text{conditional}}^{2}$	0.70	0.69	0.34	0.50

Note

*F* values are presented with *p*‐values based on Satterthwaite approximation for *df*.

Significant effects at the *p* < 0.05 level are highlighted in bold and at the *p* < 0.1 level are underlined.

Variance explained by each model is given by marginal *R* ^2^ for the fixed effects only and conditional *R* ^2^ for fixed and random effects.

Abbreviations: F, flushed; LMM, linear mixed model; N, nutrient enriched; n.a., not applicable; SRP, soluble reactive phosphorus; TN, total nitrogen; TP, total phosphorus; W, warmed.

### 2.3 Sampling

Water samples were taken once every 4 weeks (regular sampling). During extreme rainfall events, additional samples were collected immediately after the event, 1 week after the event and 3 weeks after the event, before returning to a four‐weekly schedule (Figure 1). Samples were collected using a 1 m long plastic tube which integrated the whole water column. Water samples were thoroughly mixed before further processing.

#### 2.3.1 Abiotic measurements

Total phosphorus and TN concentrations were measured following Johnes and Heathwaite (1992) and nitrate and SRP concentrations were measured following Mackereth, Heron, and Talling (1978). Photosynthetically active radiation was measured every minute by sensors located 40 cm horizontally and vertically (mid‐depth) within each mesocosm.

#### 2.3.2 Biotic measurements

##### Phytoplankton and cyanobacterial abundance

Chlorophyll‐*a* concentration (μg/L) was used as an estimate of total phytoplankton biomass. Known volumes (0.03–1 L, depending on the mesocosm) of the integrated water samples were filtered onto Whatman GF/C filters. Concentrations of the pigment were determined spectrophotometrically after cold ethanol extraction (96%) in darkness overnight (Jespersen, 1987); absorption was measured at 750 nm and at 665 nm.

The proportion of total chlorophyll‐*a* (μg/L) assigned to cyanobacteria was measured using a submersible fluorometer (bbe Moldaenke AlgaeTorch), which measured the fluorescence of phycocyanin, a quantitative biomarker for cyanobacteria. Cyanobacteria chlorophyll‐*a* (μg/L) estimated from the AlgaeTorch and cyanobacteria biovolume (mm^3^/L) estimated from microscope counts and measurements (subset of the sampling dates) were positively correlated (*R* ^2^ = 0.73, *p* < 0.001). Measurements of cyanobacteria chlorophyll‐*a* began in November 2014; in some mesocosms, chlorophyll‐*a* concentrations exceeded the calibrated range of the fluorimeter but because of an error at the user interface of the AlgaeTorch, these exceedances were undetected until the start of May 2015. Data prior to May are presented (for measurements below the manufacturer's threshold of 200 μg/L) and are discussed but are not statistically analysed. From 5 May onwards, mesocosms with chlorophyll‐*a* concentrations that exceeded the manufacturer's threshold (200 μg/L) were diluted with deionised water before measurement.

##### Phytoplankton species composition

Phytoplankton composition was identified and enumerated, from Lugol's fixed samples, the week before and 3 weeks after the flushing event in May 2015 and August 2015 using the Utermöhl technique (CEN, 2004; Utermöhl, 1958). This was done to assess the longer term effects of flushing on cyanobacteria composition—are genera with different functional traits more affected by flushing events than others? Spring and summer flushing dates were selected due to cyanobacteria bloom occurrence and because flushing events are predicted to increase during this period. For each sample, at least 400 phytoplankton units (single cell, filament or colony) were counted according to phytoplankton size classes in the whole chamber (×10), in transects (×100) or fields of view (×400 and occasionally ×630 for pico cyanobacteria). A minimum of 10 measurements of key geometric dimensions were measured for each species from images taken with a digital camera (AxioCam MRc) attached to a Zeiss Axiovert 40 CFL inverted microscope using Zen software (2012; blue edition) version 1.1.2.0. The mean of these dimensions was used to calculate biovolume (organism mm^3^/L), following Brierley, Carvalho, Davies, and Krokowski (2007). Where distinguishing features were present, organisms were identified to species, while the remainder were identified to genus, class or were unidentified.

### 2.4 Statistical analysis

Variation in chlorophyll‐*a*, cyanobacteria chlorophyll‐*a*, TP, TN, SRP, NO_3_‐N, the presence/absence of dominant cyanobacteria genera and the biovolume of dominant cyanobacteria genera were analysed with mixed models using r version 3.2.2 (R Core Team, 2017). The trend over time (for chlorophyll‐*a* and nutrients, Equations 1 and 2) and relationships with treatments (for all response variables, Equations (1), (2), (3), (4)) were tested—the fixed effects—while accounting for the random variation induced by the repeated measurements for each of the multiple mesocosms—the random effect.

To stabilise the variability, all response variables were natural log transformed, with the exception of genus presence/absence data. As a result, the assumptions of normality and homogeneity of variance were appropriate for model error terms. To account for the repeated measures within mesocosms, a random effect term was included in all models, which allowed the intercept to vary at the mesocosm level. This additional error term appropriately adjusts the coefficients and standard errors of the treatments but is also informative in quantifying additional among‐mesocosm variance, which cannot be explained by the fixed effects in the model (see, for example, Bolker et al., 2009). Autocorrelation function (AcF) plots were used to assess models for temporal autocorrelation (Hyndman et al., 2019). Models were simplified by removing non‐significant higher order interaction terms in turn. Simplified models were compared with more complex models using Akaike information criterion and Bayesian information criterion and favoured, when retaining more complex terms did not improve the model. Satterthwaite approximations of *df* were used to obtain estimated *p* values (Gaylor, 2014). The variance explained by each model is reported as marginal *R* ^2^, which describes the proportion of variance explained by the fixed factor(s) alone and conditional *R* ^2^, which describes the proportion of variance explained by both the fixed and random factors (Nakagawa & Schielzeth, 2013).

#### 2.4.1 Chlorophyll‐a and nutrients

Temporal trends in chlorophyll‐*a* and nutrient concentrations were modelled over the duration of the experiment, between July 2014 and August 2015. Linear mixed models (LMMs), using the lme4 package (Bates, Mächler, Bolker, & Walker, 2015) were used for temporal trends which could be fitted using a quadratic shape (Equation 1) while additive mixed models were used for more complex trends (Equation 2), using the gamm4 package (Wood & Scheipl, 2014), in addition to treatment covariates. Linear models were favoured as they provided greater flexibility in modelling complex interactions over time; model diagnostics were used to assess the suitability of each model. Sampling date was converted into a decimal date and mean centred (mean of zero) so that the intercept related to the mid‐point of the sampling period, mid‐February (end of the northern hemisphere meteorological winter).

1. Chlorophyll‐*a*, TP, TN and SRP

(1) $$\begin{array}{c} Y=\beta_{0}+\beta_{1}X_{\text{Time}}+\beta_{2}X_{\text{Time}}^{2}\times \delta_{\text{Nutrient}}\times \zeta_{\text{Warming}}\times \eta_{\text{Flushing}}+\gamma +\varepsilon \\ \;\;\;\gamma ∼\left(0,\sigma_{r}^{2}\right),\;\varepsilon ∼\left(0,\sigma_{r}^{2}\right) \end{array}$$

where *Y* is the response of interest; in Equations (1), (2), (3), (4), *δ*_Nutrient_, *ζ*_Warming_ and *η*_Flushing_ are the model parameters for each factor, each with two levels—current and future scenarios; *γ* is the random effect error term and *ɛ* is the overall error term—both with a mean of zero and unknown variance.

2. NO_3_

(2) $$\begin{array}{c} Y=\beta_{0}+s\left(\text{Time},\;\text{by Nutrient}\right)+\;s\left(\text{Time},\;\text{by Warming}\right)\\ \;\;+s\left(\text{Time},\;\text{by Flushing}\right)+\delta_{\text{Nutrient}}\times \zeta_{\text{Warming}}\\ \;\;\times \;\eta_{\text{Flushing}}+\gamma +\varepsilon \;\gamma ∼\left(0,\sigma_{r}^{2}\right),\;\varepsilon ∼\left(0,\;\sigma_{r}^{2}\right) \end{array}$$

where *Y* is the response (NO_3_), *s*(Time) is the smoother term for the response over time which can vary by nutrient level, warming level and flushing level (but not the interaction between these levels).

#### 2.4.2 Cyanobacteria chlorophyll‐*a*

Cyanobacteria chlorophyll‐*a* was modelled using treatment effects only (no time component, Equation (3). While time explains some additional variation (using a generalised additive mixed model), for parsimony and interpretability of the main results, the temporal trend was excluded—the response of interest was the overall effect of warming, nutrient enrichment and flushing on cyanobacteria. AcF plots and residual versus fitted plots from the final model indicated that there was no underlying temporal pattern or autocorrelation that needed to be accounted for. For comparison, as cyanobacteria is a component of the whole phytoplankton community, the relationship of chlorophyll‐*a* to the treatments was modelled for the same time period as cyanobacteria chlorophyll‐*a* (5 May–26 August).

(3) $$\begin{array}{c} Y=\beta_{0}+\delta_{\text{Nutrient}}\times \zeta_{\text{Warming}}\times \eta_{\text{Flushing}}+\gamma +\varepsilon \\ \;\;\;\varepsilon ∼\left(0,\sigma_{r}^{2}\right),\;\varepsilon ∼\left(0,\sigma_{r}^{2}\right) \end{array}$$

where *Y* is the response of cyanobacteria chlorophyll‐*a* and chlorophyll‐*a*.

#### 2.4.3 Cyanobacteria biovolume

Cyanobacteria genus biovolume data were zero‐inflated and so the analysis followed a two‐step process. First, the effect of treatment on the probability of occurrence (presence/absence) of the dominant cyanobacteria genera (*Aphanizomenon* spp.; *Microcystis* spp.; *Dolichospermum* spp.; and *Pseudanabaena* spp.) was tested using a generalised linear mixed model with a binomial distribution (Equation 4), then the effect of treatment on the biovolume of genera (for non‐zero data) was tested using an LMM (Equation 5). Sampling date, as a categorical variable (four sampling dates), was included in the model as a potential co‐variate.

1. Presence/absence of genera

(4) $$\begin{array}{c} Y=\beta_{0}+\delta_{\text{Nutrient}}\times \zeta_{\text{Warming}}\times \eta_{\text{Flushing}}+\gamma +\varepsilon \\ \;\;\;\varepsilon ∼\left(0,\;\sigma_{r}^{2}\right),\;\varepsilon ∼\left(0,\;\sigma_{r}^{2}\right) \end{array}$$

where *Y* is presence/absence data for each genus.

2. Biovolume of genera

(5) $$\begin{array}{c} Y=\beta_{0}+\omega_{\text{Sampling}}+\delta_{\text{Nutrient}}\times \zeta_{\text{Warming}}\times \eta_{\text{Flushing}}+\gamma +\varepsilon \\ \;\;\;\varepsilon ∼\left(0,\;\sigma_{r}^{2}\right),\;\varepsilon ∼\left(0,\;\sigma_{r}^{2}\right) \end{array}$$

where *Y* is the biovolume of genera (modelled individually), *ω* is the model parameter for the sampling event, with four levels: 6 May, 3 June, 29 July and 26 August 2015 (with 6 May as the intercept, reference, term).

## 3 RESULTS

### 3.1 Treatment effects on nutrient concentrations

The effects of treatments on nutrient concentrations (total and biologically available) were statistically significant over the course of the experiment (Figure 2; Table 1). The concentration of TP increased from spring (March) onwards, in particular in nutrient‐enriched mesocosms and heated mesocosms, in the latter case including both ambient‐nutrient and nutrient‐enriched mesocosms (Figure 2a). The concentration of SRP decreased from spring onwards in all treatments except for heated, nutrient‐enriched mesocosms in which concentrations increased (Figure 2b). The concentration of TN increased not only in unheated, nutrient‐enriched mesocosms but also in heated, ambient‐nutrient mesocosms (Figure 2c). During summer, spring and autumn, nitrate concentrations were low in all treatments (Figure 2d). The increase in concentrations during the winter was statistically significant in nutrient‐enriched mesocosms, and also, but to a lesser extent, in heated, ambient‐nutrient mesocosms (Table 2). Flushing had no effect on nitrate concentrations.

**Table 2:** GAMM results for log nitrate (NO_3_‐N, mg/L) response (July 2014–August 2015)

a. Parametric coefficients. Changes on the intercept (end of February) after removing non‐significant terms sequentially				
	Intercept	Nutrient enriched		
Estimate	**−3.71**	**0.67**		
b. Estimated *df* (edf) for approximately significant time smooth terms for nutrient treatment and warming treatment				
	Ambient‐nutrient	Nutrient enriched	Ambient	Warmed
edf	**6.34**	**7.61**	0.75	**6.35**

Note

Significant effects (*p* < 0.05) are highlighted in bold.

*R* ^2^ adjusted = 0.59.

Abbreviation: GAMM, generalised additive mixed model.

### 3.2 Treatment effects on total phytoplankton

**Figure 3:**
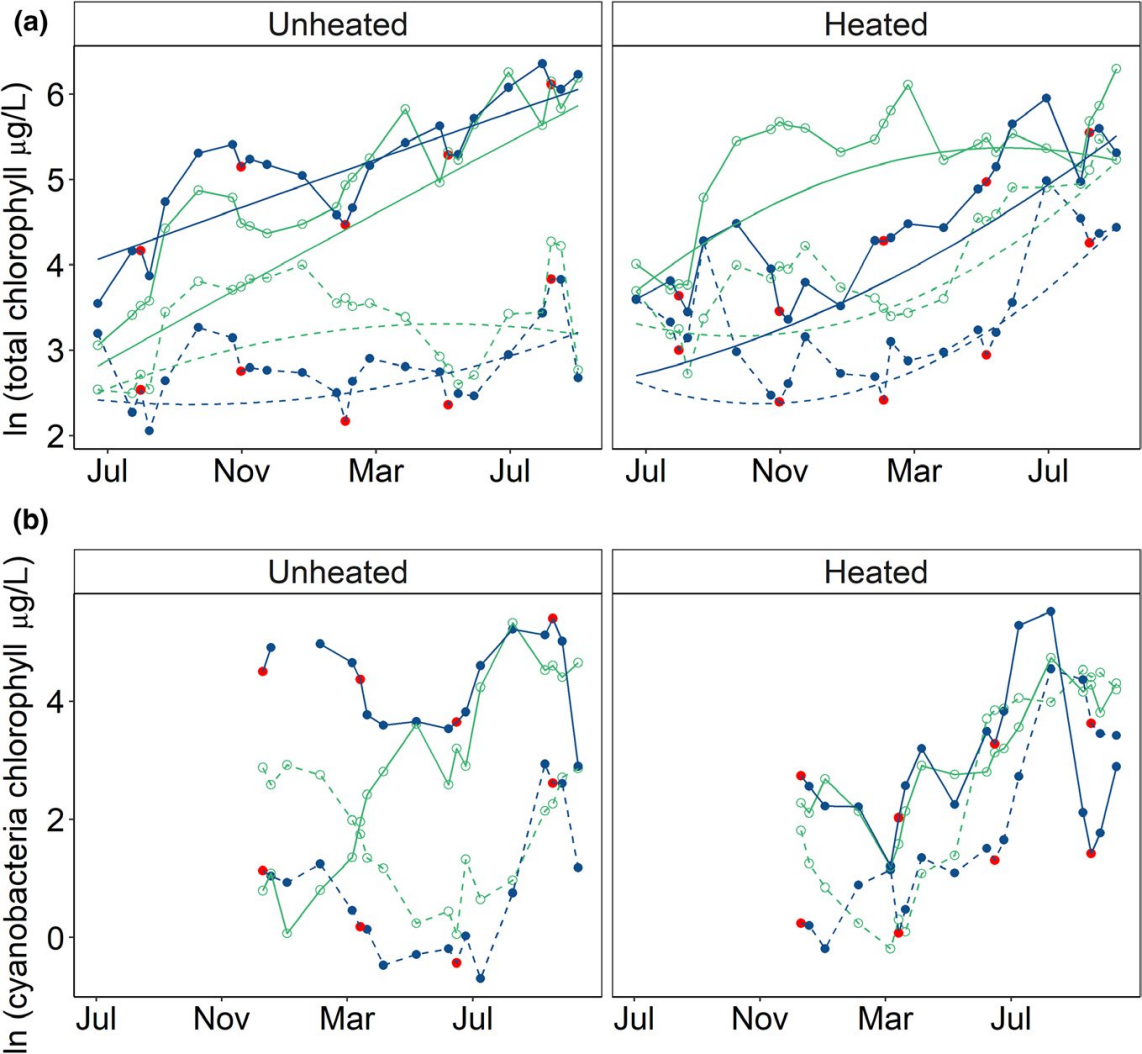
Effect of warming, nutrient addition and extreme rainfall (flushing) events on the concentration of (a) ln total chlorophyll‐*a* (μg/L) and (b) ln cyanobacteria chlorophyll‐*a* (μg/L) over the duration of the experiment. Data points are ln transformed mean responses: blue, flushed; green, unflushed; solid line, nutrient enriched; dashed line, ambient‐nutrient; left‐hand side, unheated treatments; right‐hand side, heated treatments. For flushed treatments (blue lines), red data points are sampling events the day immediately after an extreme flushing event. The smooth lines in panel (a) are the fitted response from the best fitting linear mixed model (marginal *R* ^2^ = 0.57)—Figure S6 for model confidence intervals. In panel (b), cyanobacteria chlorophyll‐*a* data are only presented qualitatively as, prior to May, in some treatments (nutrient enriched–flushed, heated–nutrient enriched and heated–nutrient enriched–flushed), replicates varied between *n* = 0 [missing data point] and *n* = 4). These data were not missing at random and so the data were not statistically modelled over this period

The concentration of chlorophyll‐*a* showed statistically significant variation over time and with treatments (Table 1; Figure 3a), with trends generally following changes in TP (Table 1; Figure 2a). Chlorophyll‐*a* concentrations increased linearly with time in unheated, nutrient‐enriched mesocosms while the response in all other treatments showed different time‐dependent responses. In heated mesocosms, the greatest increases in chlorophyll *a* occurred from around March onwards in all treatments except for in heated, unflushed, nutrient‐enriched mesocosms in which concentrations remained fairly constant from this point. After accounting for the effects of treatment and time, an additional 14% of variance was explained by between‐mesocosm differences (conditional *R* ^2^ = 0.72).

### 3.3 Treatment effects on total cyanobacteria

During the period of sampling (December 2014–August 2015), the abundance of cyanobacteria generally followed a seasonal pattern observed in many shallow, temperate lakes, with highest values in summer (Figure 3b). However, in nutrient‐enriched, flushed mesocosms, cyanobacterial dominance and abundance extended beyond the typical season (Figure 3b). In this treatment, on average, 55% of winter (December 2014–February 2015) phytoplankton abundance was accounted for by cyanobacteria, while the average percentage cyanobacteria during the same period in all other treatments was 15 ± 14%.

**Figure 4:**
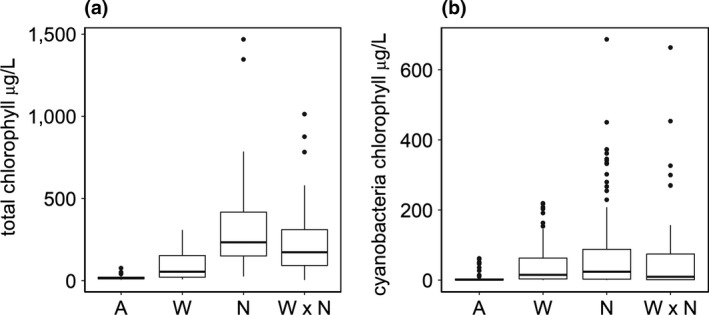
Total chlorophyll‐*a* (μg/L) (a) and cyanobacteria chlorophyll‐*a* (μg/L) (b), between 5 May and 26 August 2015. Data are plotted by the statistically significant treatment effects from the best fitting linear mixed model for each response (Table 3). A, ambient (unheated and ambient‐nutrient); N, nutrient enrichment only; W, warming only (heated mesocosms); WN, warming and nutrient enrichment together. The lower and upper hinges correspond to the 25th and 75th percentiles, the whiskers extend to 1.5 times the interquartile range

The abundance of cyanobacteria, between May 2015 and August 2015, was explained by a negative interaction between warming and nutrient enrichment. Warming and nutrient enrichment, as single stressors, resulted in statistically significantly higher cyanobacteria than in ambient (unheated, ambient‐nutrient) mesocosms. However, in combination, these stressors dampened the effect of one another, resulting in a weak antagonism (negative interaction), that is the response (abundance) was less than the combined (additive) effect of warming and nutrient enrichment as single stressors. Increases in cyanobacteria, the size effect, in response to warming and nutrient enrichment as single stressors were similar (Figure 4b; Table 3). During the same period, the abundance of total phytoplankton was also explained by a negative interaction between warming and nutrient enrichment. Total phytoplankton was less sensitive to warming than cyanobacteria, that is total abundance increased more in response to nutrient enrichment, as a single stressor, than warming (Figure 4a; Table 3).

**Table 3:** LMM coefficients (±*SE*) for ln total chlorophyll‐*a* (μg/L) and ln cyanobacteria chlorophyll‐*a* (μg/L) relationship to treatments between May and August 2015

	Intercept	N	W	N × W	$R_{m}^{2}$	$R_{c}^{2}$
ln total chlorophyll‐*a* (μg/L)	**3.0 ± 0.2**	**2.6 ± 0.3**	**1.1 ± 0.3**	**−1.6 ± 0.4**	0.53	0.67
ln cyanobacteria chlorophyll*‐a* (μg/L)	0.2 ± 0.6	**2.5 ± 0.8**	**2.5 ± 0.8**	**−3.4 ± 1.1**	0.15	0.43

Note

Significant effects (*p* < 0.05) are highlighted in bold.

The variance explained by each model is given by marginal $R_{m}^{2}$ for the fixed effects only and conditional $R_{c}^{2}$ for the fixed and random effects.

Abbreviation: LMM, linear mixed model.

Flushing events resulted in statistically significant reductions in total phytoplankton and cyanobacterial abundance (Table S3); however, these losses did not have long‐term effects on total phytoplankton or cyanobacterial abundance, that is flushing as a treatment did not explain additional variation in the abundance of total phytoplankton or cyanobacteria sampled between May and August, indicating that recovery was rapid during the spring/summer months.

After accounting for the effects of treatment, an additional 28% and 14% of variance was explained by between‐mesocosm differences for the response of cyanobacterial chlorophyll‐*a* and total chlorophyll‐*a* respectively.

### 3.4 Treatment effects on the composition of cyanobacteria

Cyanobacterial biovolume was mainly composed of nitrogen‐fixing cyanobacteria (68%), in particular *Aphanizomenon* spp. (51%, 3.64 × 10^8^ mm^3^/L) but also *Dolichospermum* spp. (17%, 1.15 × 10^8^ mm^3^/L). Other notable contributions to cyanobacterial composition were from *Microcystis* spp. (13%, 8.41 × 10^7^ mm^3^/L) and *Pseudanabaena* spp. (13%, 8.47 × 10^7^ mm^3^/L; (Figure S7; Table S4).

**Figure 5:**
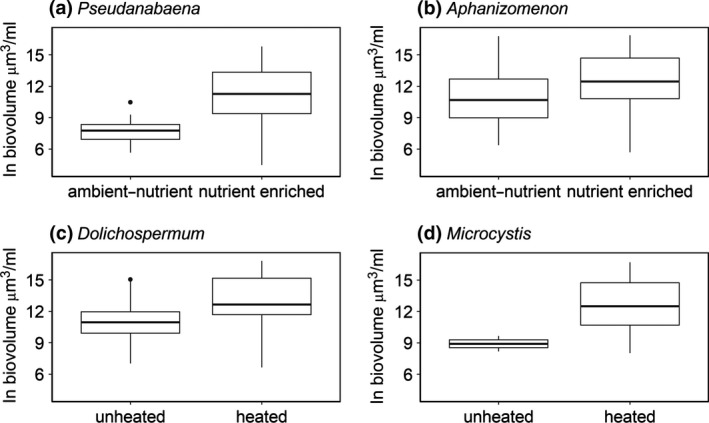
Natural log biovolume (μm^3^/ml) of the dominant genera of cyanobacteria—(a) *Pseudanabaena *spp.; (b) *Aphanizomenon *spp.; (c) *Dolichospermum *spp.; (d) *Microcystis *spp.—observed in May, June, July and August 2015, plotted by the statistically significant treatment effects from the best fitting linear mixed model for each genus. The solid line corresponds to the median, the lower and upper hinges correspond to the 25th and 75th percentiles, the whiskers extend to 1.5 times the interquartile range

At the genus level, the occurrence and abundance of the dominant genera—*Aphanizomenon* spp., *Dolichospermum* spp., *Microcystis* spp. and *Pseudanabaena* spp. (Table 4)—were explained by single stressor effects only, that is no statistically significant interactive effects of stressors were detected. *Aphanizomenon* spp. was fairly ubiquitous, although its abundance was statistically significantly higher in nutrient‐enriched mesocosms (Table 4; Figure 5b). *Dolichospermum* spp. occurrence was statistically significantly higher in nutrient‐enriched mesocosms and in samples taken later in the summer (July and August) while biovolume was statistically significantly higher in heated mesocosms (Table 4; Figure 5c). *Microcystis* spp. occurrence and biovolume was strikingly related to warming: 94% of occurrences were in heated mesocosms, although overall this genus was only present in 25% of the samples. The occurrence of *Microcystis* spp. also depended on the time of the year, with statistically significantly higher occurrence during July and August compared to May (Table 4; Figure 5d). The occurrence and abundance of *Pseudanabaena* spp. was positively explained by nutrient enrichment (Table 4; Figure 5a).

**Table 4:** Summary (coefficient and *SE*) of best fit GLMMs explaining the probability of the presence of dominant cyanobacteria taxa (present/absent) and LMMs explaining taxa biovolume (natural log mm^3^/L), when present—that is non‐zero data. The variance explained by each model is given by marginal $R_{m}^{2}$ for the fixed effects only and conditional $R_{c}^{2}$ for the fixed and random effects. Sampling date is a factor (*n* = 4): 6 May, 3 June, 29 July and 26 August 2015. In models where date is significant, the intercept relates to 6 May and all other levels of date are compared to data from this date

Taxa	Intercept	Warming	Nutrient enrichment	Warming × nutrient enrichment	Sampling date			$R_{m}^{2}$	$R_{c}^{2}$
					3 June	29 July	26 August		
Nostocales									
Presence	**1.50 ± 0.32**							0.00	0.16
Biovolume	**11.10 ± 0.57**	**2.15 ± 0.76**	**2.59 ± 0.79**	**−2.42 ± 1.12**				0.13	0.18
*Aphanizomenon*									
Presence	**0.62 ± 0.27**							0.00	0.22
Biovolume	**10.89 ± 0.52**		**2.30 ± 0.72**					0.15	0.30
*Dolichospermum*									
Presence	−1.1 ± 0.66		**−2.41 ± 0.78**		**1.43 ± 0.73**	**3.05 ± 0.83**	**2.43 ± 0.83**	0.36	0.58
Biovolume	**11.10 ± 0.56**	**1.72 ± 0.75**						0.11	0.29
Other genera									
*Microcystis*									
Presence	**−9.57 ± 2.72**	**7.27 ± 2.33**			0.57 ± 1.09	**2.53 ± 1.17**	**4.94 ± 1.63**	0.36	0.68
Biovolume	12.17 ± 0.63							0.00	0.58
*Pseudanabaena*									
Presence	**−1.37 ± 0.31**		**1.49 ± 0.40**					0.15	0.15
Biovolume	**7.77 ± 0.78**		**3.45 ± 0.95**					0.26	0.51

Note

Significant effects (*p* < 0.05) are highlighted in bold; blanks signify that these terms did not significantly improve the model.

Abbreviation: GLMM, generalised linear mixed model; LMM, linear mixed model.

At a higher taxonomic grouping, statistically significant treatment interactions were detected for biovolume of the group Nostocales (*Aphanizomenon* spp. and *Dolichospermum* spp.). The response at this higher grouping reflects the results obtained at the cyanobacterial community level (cyanobacterial chlorophyll‐*a*) with positive effects of nutrient enrichment and warming alone and a negative interaction together. Most *Aphanizomenon* spp. and *Dolichospermum* spp. filaments contained specialised heterocyte cells that are involved in the fixation of nitrogen (Figure 5).

## 4 DISCUSSION

Climate change is often studied as a single stressor (warming) impacting on the natural environment. In reality, this masks a great deal of complexity (e.g. Paerl et al., 2016) with changes in the timing and extremity of weather events often ignored in favour of responses to general climate trends that are more straightforward to analyse. Experimental mesocosm studies offer an approach to investigate this complexity in order to develop a clearer mechanistic understanding of the interactions between multiple stressors, allowing quantification and comparison of individual stressor effects and their interactions (Crain, Kroeker, & Halpern, 2008; Feuchtmayr et al., 2009; Piggott, Salis, Lear, Townsend, & Matthaei, 2015).

### 4.1 The effects of nutrient enrichment

Despite high nutrient concentrations in the ‘current’ nutrient (average of 100 µg/L TP) and ‘future’ nutrient (average of 314 µg/L TP) scenarios, it is interesting that phytoplankton and cyanobacterial abundance showed clear increases in response to the high nutrient loading treatment (in the absence of other stressors). This is worth noting because of the widely reported asymptotic behaviour of chlorophyll‐*a* and cyanobacteria to TP (Carvalho et al., 2013; McCauley, Downing, & Watson, 1989; Phillips et al., 2008; Watson, McCauley, & Downing, 1992), with ~100 µg/L being a typical turning point. This suggests that nutrient controls of phytoplankton abundance can occur in very nutrient‐enriched systems when other factors do not limit the response such as grazing (Yvon‐Durocher et al., 2015). The response to nutrient enrichment in these mesocosms was likely not inhibited by grazing pressures because of the presence of planktivorous fish and the observed absence of large zooplankters.

### 4.2 The effects of warming

The effects of warming on total phytoplankton abundance and cyanobacterial abundance depended on nutrient loading, while the effects of warming varied between cyanobacteria genera, and was independent of nutrient loading and flushing.

#### 4.2.1 Total phytoplankton and total cyanobacterial abundance

In ambient‐nutrient mesocosms, as expected, warming increased the abundance of cyanobacteria and phytoplankton. The overall increase in phytoplankton abundance in these mesocosms can be explained by direct effects of temperature on growth rates (Reynolds, 2006), but increases in total standing crop are more likely to be due to indirect effects of temperature on the availability of nutrients needed for growth, due to release of phosphorus from the sediment (Jensen & Andersen, 1992)—this was not directly measured but was inferred from a mass balance calculation. The latter process, at least, seemed to be important from late spring into the summer in heated mesocosms (Figure 2a). The direct effect of temperature on growth rates was particularly important for the abundance of cyanobacteria, for which we observed statistically significant increases in common bloom‐forming taxa, *Microcystis* spp. and *Dolichospermum* spp.—the maximum growth rates of these genera are generally reached at higher temperatures compared to other cyanobacteria and phytoplankton taxa (e.g. Lürling et al., 2017). This result supports the expectation that changes in water temperature will drive shifts in phytoplankton composition, with higher temperatures not only favouring cyanobacteria in general, but, particularly those genera that commonly form dense blooms in freshwaters and are known toxin producers (De Senerpont et al., 2007; Jöhnk et al., 2008). It should be emphasised that these effects of warming were observed in nutrient‐rich systems. The stimulatory effect of temperature depends on the carrying capacity of the system (Elliott, 2012; Lürling et al., 2017), thus warming will have less potential to increase biomass in sites with low nutrient availability. Unexpectedly, we found that warming in combination with high nutrient enrichment reduced the abundance of cyanobacteria. This result is striking because it contrasts with the widely hypothesised (Paerl & Huisman, 2008) and observed (Lürling et al., 2017; Rigosi, Carey, Ibelings, & Brookes, 2014; Taranu et al., 2012) synergistic interaction between warming and nutrient enrichment on cyanobacterial abundance. However, it is important to emphasise that the effect size of this interaction was small in this study. Importantly, even though cyanobacterial and total phytoplankton abundance were less than the expected additive effect, they were still higher than the ambient (control) mesocosms (Figure 4) and resulted in cell densities that exceed World Health Organization threshold guidelines for drinking and bathing waters (Chorus & Bartram, 1999). The mechanism for the antagonism is unclear but is probably linked to the high productivity of the mesocosms as a result of high nutrient loading. An antagonism between warming and nutrient enrichment was also detected for total phytoplankton, indicating that another factor(s) was limiting phytoplankton growth. Under these conditions, and in contrast to all other treatments, SRP was plentiful indicating that phosphorus limitation was not responsible for the observed response. Nitrogen and light limitation are also excluded as mechanisms since nitrate concentrations were similar (and low) in all treatments during the summer and light attenuation was no higher in warmed, high nutrient addition mesocosms than in high nutrient addition mesocosms (Figures S7 and S8). One explanation could be depletion of dissolved inorganic carbon, that can lead to carbon limitation (Jansson, Karlsson, & Jonsson, 2012), which has been shown to occur under nutrient‐enriched conditions (Maberly, 1996) and which may be exacerbated by warming (Yvon‐Durocher, Hulatt, Woodward, & Trimmer, 2017). Unfortunately, available carbon was not directly measured, nor could it be estimated from the available data, and so this explanation cannot be fully tested. Some insight into carbon availability can be gained from differences in pH among treatments; high pH episodes occurred during the summer in nutrient‐enriched mesocosm indicating that carbon availability was likely to be low in these mesocosms, yet pH was no higher in heated mesocosm than in unheated mesocosms (Figure S10). Lower CO_2_ dissolution with increasing temperature could further reduce carbon availability in heated mesocosms.

In other lake types, or at different levels of nutrient or temperature stress, other interaction effects are possible between multiple stressors. For example, synergistic responses of cyanobacteria may occur in lakes with greater grazing pressure because: (a) lower productivity will relieve other limitations on growth (as seen in this study); and (b) cyanobacteria could benefit because of resistance to grazing (Gliwicz, 1990; Lampert, 1987). Different responses may also arise depending on the extent of nutrient loading (Piggott et al., 2015; Rigosi et al., 2014). Synergies may be possible along oligotrophic to mesotrophic parts of the TP gradient where TP–chlorophyll‐*a* and TP–cyanobacteria relationships are strongest and linear (Carvalho et al., 2013; Phillips et al., 2008; Richardson et al., 2018). Others report synergies when analysing the dominance (proportion) of cyanobacteria in eutrophic or hypertrophic systems, which they argue, proportionally, favour cyanobacteria over other phytoplankton (Rigosi et al., 2014). Finally, interaction strengths can depend on lake type (Taranu et al., 2012). This study contributes to the growing evidence that environmental context is important to understand and predict the influence of multiple stressors on the prevalence of algal abundance, particularly potentially harmful cyanobacterial blooms.

#### 4.2.2 Composition

The antagonistic effects of warming and nutrient enrichment were only detected at the community level (total cyanobacteria). At the genus level, no statistically significant treatment interactions were found; rather warming resulted in the increased abundance of *Dolichospermum* spp. and *Microcystis* spp. and nutrient enrichment resulted in the increased abundance of *Aphanizomenon* spp. and *Pseudanabaena* spp. Differences in the sensitivity of genera to anthropogenic stressors have been found before (Ekvall et al., 2013; Rigosi et al., 2014), and should be expected as cyanobacteria are a diverse group with a wide range of eco‐physiological characteristics that will lead to varying responses (Carey et al., 2012; Mantzouki et al., 2016). Differences in community and population level responses to multiple stressors have also been found for a variety of other biological groups (Côté, Darling, & Brown, 2016; Crain et al., 2008).

### 4.3 The effects of flushing events

It is expected that general recovery of phytoplankton biomass following losses from flushing will depend on factors that limit growth; these may be influenced naturally by seasonal effects on growth‐limiting factors, such as light and temperature, or influenced by anthropogenic pressures on the system, such as land use (nutrient loading) and climate change, for example warming. In spring and summer, flushing had short‐term effects on phytoplankton abundance in all treatments while in autumn and winter, the effects were more prolonged (longer recovery). It is likely that the conditions for growth during spring and summer were suitable in all treatments to allow for rapid recovery while seasonally limiting factors such as light and temperature prolonged recovery outside of the main growing season. Enhanced nutrient loading did not confer any additional benefit in the recovery from flushing, probably because no additional nutrients were added to simulate increased loading with run‐off (Reichwaldt & Ghadouani, 2012). In real‐world flushing events, a set of different nutrient‐loading scenarios (decrease, stasis, increase) are possible depending on factors such as the season in which the event occurs (Donohue, Styles, Coxon, & Irvine, 2005) and the source of nutrients—point or diffuse (Elliott et al., 2009).

Cyanobacteria are sensitive to high flushing rates (Carvalho et al., 2011; Reynolds, Huszar, Kruk, Naselli‐Flores, & Melo, 2002), yet flushing had no effect on cyanobacteria in the mesocosms. This may be explained by: (a) the limited frequency of the events; (b) the short duration of the events; (c) the timing of the events; and (d) the extent of disruption to the physical environment. Cyanobacterial dominance and abundance are observed to be suppressed in lakes with a greater frequency of disruptions (Padisák et al., 1999; Wood et al., 2017). This relates to the intermediate disturbance hypothesis (Connell, 1978), with infrequent disturbance resulting in competitive exclusion. At three monthly intervals, the flushing events were likely too infrequent in the mesocosms for the competitive exclusion of cyanobacteria. For example, Padisák et al. (1999) found that *Aphanizomenon* blooms were supressed when flushing rates were increased to a frequency of every 20–30 days. Cyanobacteria are also usually suppressed in highly flushed lakes (short retention time systems) as high flow selects for faster growing taxa like diatoms (Cross, McGowan, Needham, & Pointer, 2014; Dickman, 1969; Sherman, Webster, Jones, & Oliver, 1998), thus preventing the system from expressing its full trophic potential (Reynolds et al., 2012). It is likely that the duration of the flushing events in the mesocosms was too short for the sensitivities of different phytoplankton to flushing to become apparent (Reynolds et al., 2002). The timing of perturbation is also an important factor in the response of phytoplankton communities, because of inherent seasonal variation in phytoplankton abundance and composition (Sommer et al., 2012; Sommer, Gliwicz, Lampert, & Duncan, 1986) and consequent seasonal sensitivities and tolerances of impacted communities (Connell, 1978). Functional groups with tolerances to nutrient segregation and thermal stability but sensitivities to mixing and flushing such as *Dolichospermum* and *Microcystis* are expected to be more sensitive to flushing during the summer months when they most often dominate (Elliott, 2010; Verspagen et al., 2006). It is interesting then that flushing increased cyanobacterial abundance and dominance in nutrient‐enriched mesocosms during the winter months. Although uncommon, winter blooms of cyanobacteria can occur naturally, comprising taxa such as *Planktothrix rubescens* (Naselli‐Flores, Barone, Chorus, & Kurmayer, 2007) and *Aphanizomenon* spp. (Reynolds et al., 2002), which are efficient at growing under low light conditions. Thus the response of the phytoplankton community to flushing may depend on seasonal timing in ways not previously expected. Other changes in selection pressures that affect community composition, such as destratification, changes in water colour and increased turbidity, were not tested in the scope of this study. This study shows that flushing‐induced loss of biomass, in the absence of other change, does not have long‐term effects on phytoplankton abundance or composition when events occur at times of the year when light and temperature are not limiting. Future experiments could investigate more specific scenarios of hydrological change, such as the ‘perfect storm’ of a large pulse of nutrients followed by a dry period of no flushing (Paerl et al., 2016), or combinations of other hydrological factors, such as increased turbidity/changes in colour, to determine the importance of hydrology in isolation and in combination.

### 4.4 Management implications

Our results suggest that under future climate and nutrient scenarios, nutrients may need to be substantially reduced in shallow lakes in order to: (a) mitigate against the indirect effects of warming‐enhancing nutrient cycling, especially in previously impacted lakes; and (b) mitigate against the direct effects of enhanced growth rates of common bloom‐forming species of cyanobacteria, that are widely recognised for their potential to produce harmful toxins (Codd et al., 2005).

It should be stressed that context is important, so the combined effects of warming, nutrient enrichment and flushing events on phytoplankton and cyanobacterial abundance observed in this study are relevant to enriched, shallow lakes. Effects of climate change are likely to differ between shallow and deep lakes (Richardson et al., 2018). In shallow lakes, as observed in our shallow mesocosms, warming may benefit cyanobacteria through enhanced internal loading of P (Dolman, Mischke, & Wiedner, 2016; Søndergaard, Lauridsen, Johansson, & Jeppesen, 2017) and potential increased benefits for N‐fixers caused by increased denitrification rates (Veraart, Klein, & Scheffer, 2011) while in deeper lakes, the benefits may emerge because of increased stability in the physical structure of the lake (Taranu et al., 2012). In shallow lakes, flushing events are likely to affect phytoplankton loss rates and alter nutrient concentrations more than deep lakes, but in deep lakes, flushing events may impact through destratification (Sherman et al., 1998). Food web interactions are also important system factors to consider in shaping the response of phytoplankton to environmental change. Both top‐down grazing pressures (Yvon‐Durocher et al., 2015) and the effects of macrophytes (Feuchtmayr et al., 2009; McKee et al., 2003; Moss et al., 2003) can be important in individual lakes. Mesocosm experiments may alter some of these processes through enclosed container effects, although sufficient replication of treatment versus control mesocosms should ensure these container effects are taken into account.

### 4.5 Final remarks and future studies

This study builds a foundation for understanding the complexity of how global climate change may impact on freshwater resources. It highlights the clear need to mitigate against global warming, but indicates that ecological surprises may occur depending on the lake characteristics and stressor context (e.g. low or high nutrient loading, season). Consequently, oversimplification of global change effects on cyanobacteria should be avoided; stressor gradients and seasonal factors should be considered important in predicting the response. Future studies should test other ‘real‐world’ possibilities of different nutrient scenarios and other hydrological changes, such as more extreme droughts, which, following extreme rainfall events, are likely to benefit cyanobacteria growth and bloom dynamics (Paerl et al., 2016).
