# Supplementary material for: Response of cyanobacteria and phytoplankton abundance to warming, extreme rainfall events and nutrient enrichment
Source: Glob Chang Biol. 2019 Jul 4;25(10):3365–80. doi: 10.1111/gcb.14701 (PMC6852574; doi:10.1111/gcb.14701)
Supplement: Supplementary file 1 [file GCB-25-3365-s001.zip › gcb14701-sup-0001-Supinfo.docx]

**Supplementary material**

### **Supplementary methods**

Estimates of nutrient loss through extreme rainfall events.

To calculate the amount of nutrients lost during flushing events we used the following formula to calculate the dilution curve:

$$\frac{nutrient concentration at time n (minutes)}{initial volume} \times\left( initial volme-flow rate \right)$$

in which the initial volume was 3000 L and the flow rate was calculated from the time it took to pump 1500 L of water into the mesocosm; we used two different pumps, one with a flow rate of 71 L minute^-1^ and the other with a flow rate of 100 L minute^-1^. Nutrient concentrations were calculated after one minute of the given flow rate from initial nutrient concentrations (μg mL^-1^) within the mesocosm, these updated concentrations were then used to iteratively calculate the concentration after each successive minute until the total minutes of flushing was reached for each respective pump (15 minutes and 21 minutes). Initial nutrient concentrations were estimated from the preceding sampling event. The difference in nutrient concentrations between the initial concentration and the concentration lost was replaced to each treated mesocosm (after subtracting nutrient concentrations recorded in the water used for flushing).

Light attenuation analysis

A potential explanation for the antagonistic interaction between warming and nutrient enrichment is that potential treatment effects on phytoplankton composition could result in greater self-shading. To explore this we explored potential differences in light attenuation among treatments.

A light attenuation coefficient (k m^-1^) was calculated from mean daily measurements of PAR (photosynthetically active radiation) using the following equation:

$$k m^{-1}=\frac{\ln\left( \frac{\mathrm{PAR}_{air}}{\mathrm{PAR}_{mesocosm}} \right)}{0.45}$$

PAR was recorded every minute by sensors located 40cm horizontally and vertically (mid-depth) within each mesocosm. K was higher in nutrient enriched mesocosms (Fig. S8, S9), in which attenuation from algae was higher (because of higher biomass in these mesocosms, main text Fig. 2-3) however K was no higher in warmed mesocosms than ambient mesocosms or in warmed x nutrient enriched mesocosms. This indicates that the mechanisms of the antagonism is not light limitation through increased self-shading.

### **Supplementary figures**

| 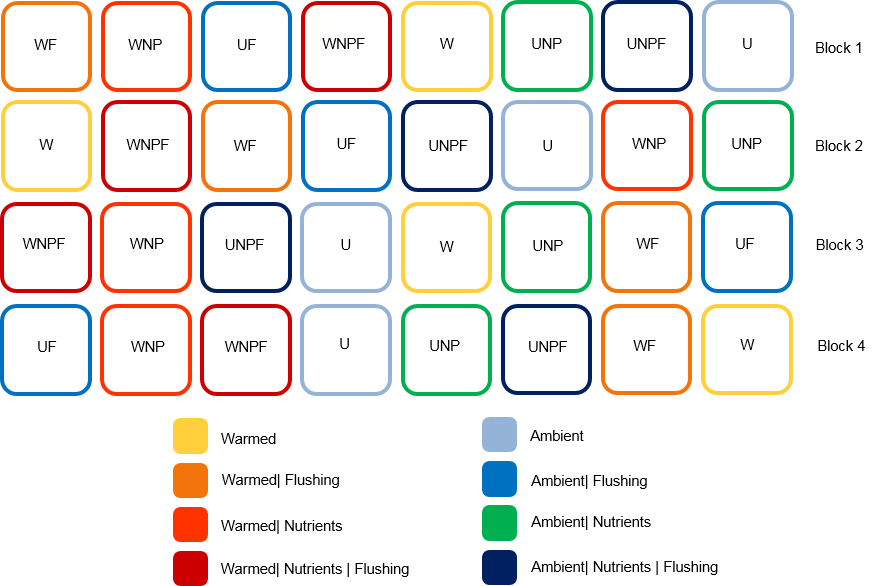 |
| --- |
| Fig. S1 Schematic of the experimental set-up. There were eight treatments in total, as represented by the different colours. Each treatment was repeated four times, one replicate randomly assigned to a mesocosm in each experimental block. U = unheated/ambient, W = warmed, N = nutrient enriched, F = flushed e.g. UNPF = unheated, nutrient enriched, flushed. |

| 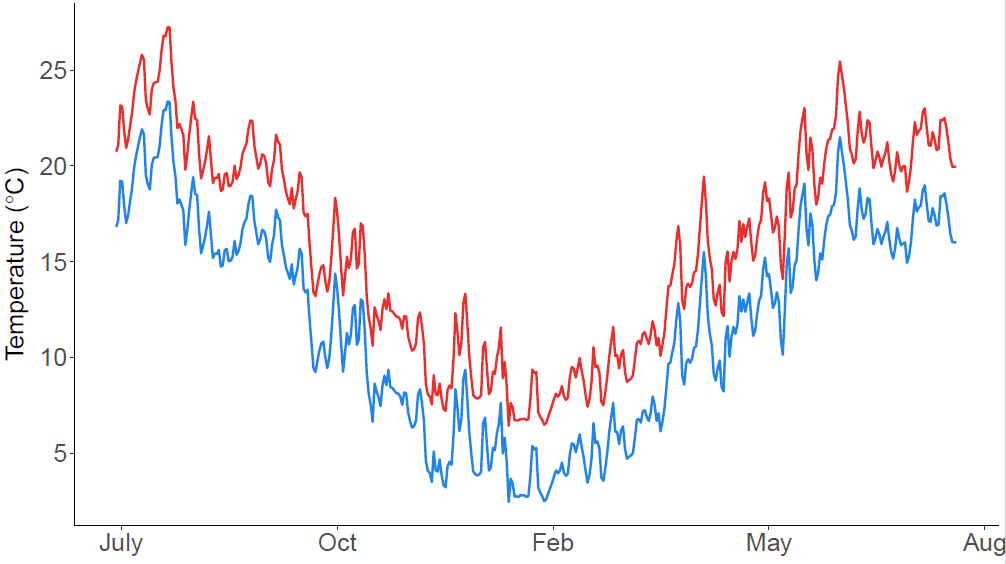 |
| --- |
| Fig. S2 Mean daily water temperatures (°C) in mesocosms between July 2014 and August 2015 in 16 mesocosms at ambient temperature (blue) and 16 mesocosms warmed to 4°C above ambient (red). |


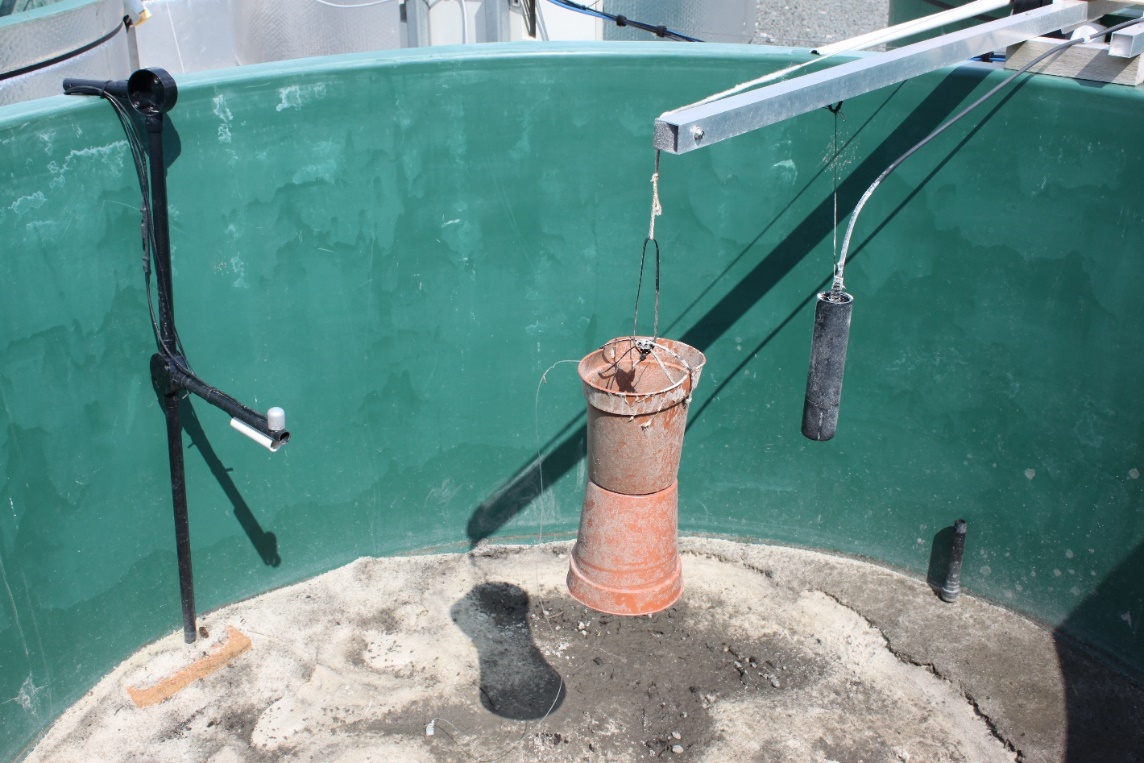


Fig. S3 Mixers are suspended in the middle of each mesocosm and move up and down to allow disruption of thermal stratification. The top and bottom of the mixers are hollow to increase mixing.


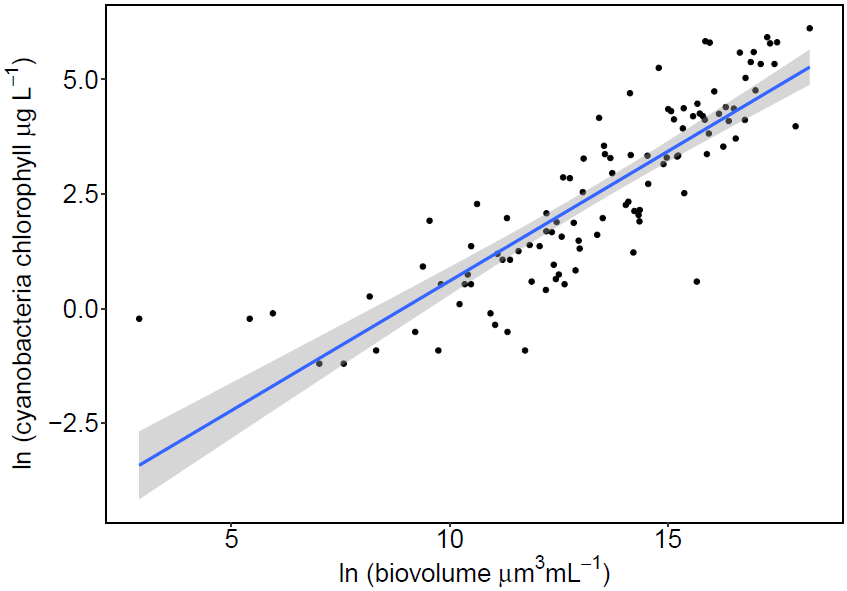


Fig. S4 Relationship between natural log cyanobacteria biovolume (microscope counts and measurements) and natural log cyanobacteria chlorophyll-*a* (measured using the AlgaeTorch).


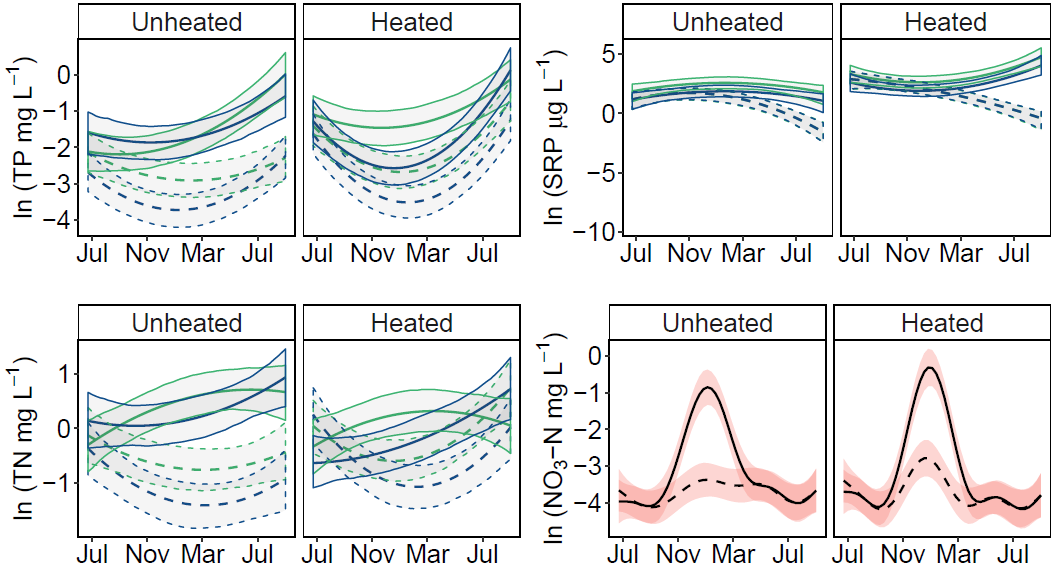


Fig. S5 Effect of nutrient enrichment and extreme rainfall events on the concentration of (a) TP (marginal R^2^ = 0.55), (b) SRP (marginal R^2^ = 0.29), (c) TN (marginal R^2^ = 0.37) and (d) NO_3_-N (R^2^ adjusted = 0.59) in ambient and warmed mesocosms over time (July 2014 – August 2015). Smooth lines in panels (a – c) are the predicted fitted responses from the best fitting LMM model (Table 1): blue, flushed; green, unflushed; solid line, nutrient enriched; dashed line, ambient-nutrient. The smooth black lines in panel (d) are predicted fitted responses from the best fitting AMM (Table 2): solid line, nutrient enriched; dashed line, ambient-nutrient addition. Shaded areas are bootstrapped 95% confidence intervals for the model.


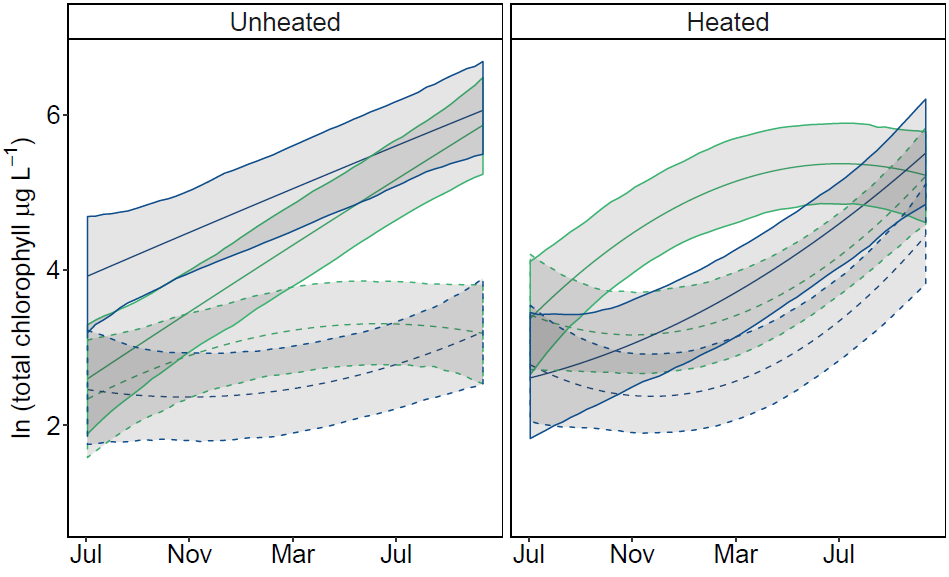


Fig. S6 Effect of warming, nutrient addition and extreme rainfall (flushing) events on the concentration of ln total chlorophyll-*a* (μg L^-1^). The smooth lines in are the fitted response from the best fitting LMM (marginal R^2^ = 0.57): blue, flushed; green, unflushed; solid line, nutrient enriched; dashed line, ambient-nutrient; left hand side, unheated treatments; right hand side, heated treatments. Shaded areas are bootstrapped 95% confidence intervals for the model.

| 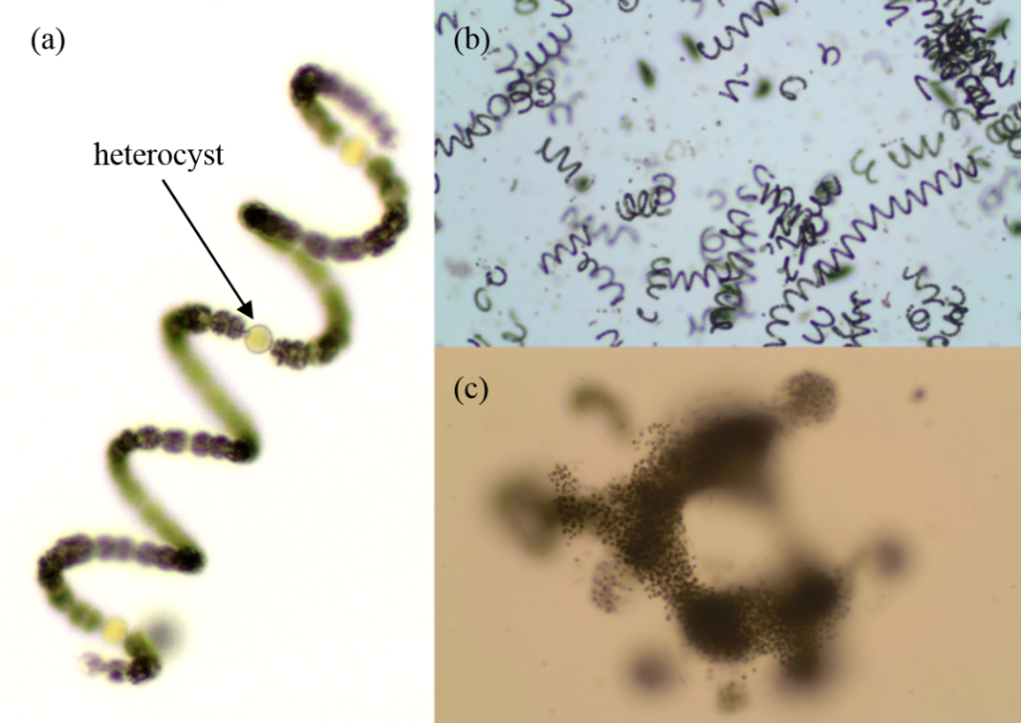 |
| --- |
| Fig. S7 Images of some of the dominant cyanobacteria genera observed between the 5^th^ of May and 26^th^ of August 2015. Image (a) and (b) are *Dolichospermum* spp.; (b) shows the high density of individual filaments seen within some of the samples; (c) shows a colony of *Microcystis* sp. |


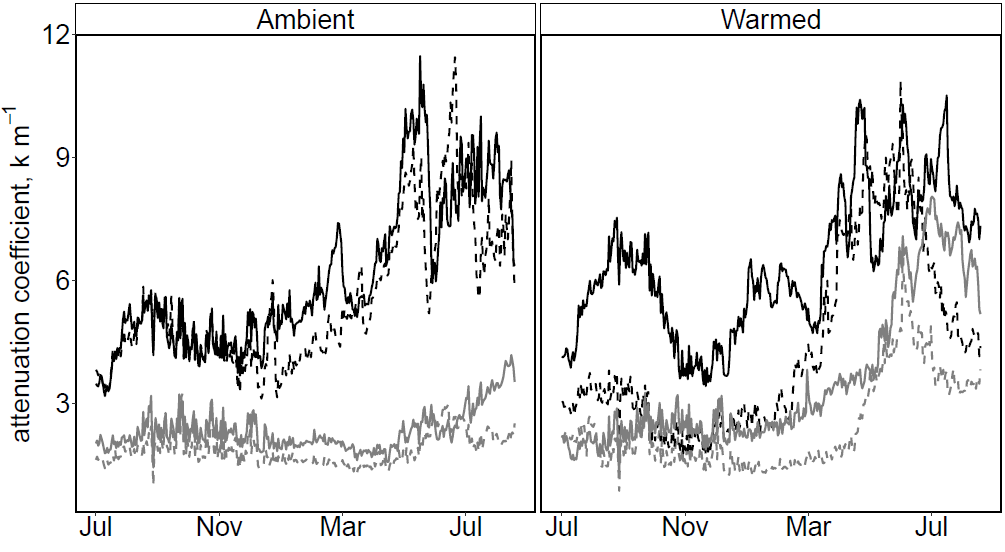


Fig. S8 Mean daily light attenuation coefficient (k m^-1^) in different treatments over the duration of the experiment. Solid line, unflushed; dashed line, flushed; black line, nutrient enriched; grey line, ambient-nutrients; left hand side, ambient temperature; right hand side, warmed.


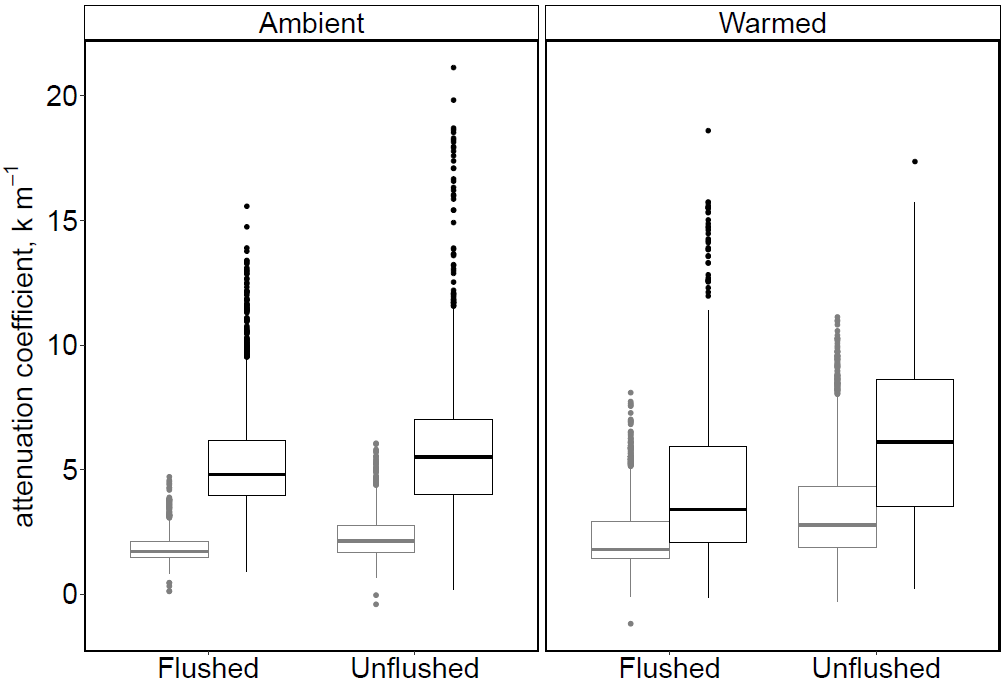


Fig. S9 Boxplot of mean daily attenuation of light (k m^-1^) in different treatment. Black, nutrient enriched; grey, ambient-nutrients. The lower and upper hinges correspond to the 25^th^ and 75^th^ percentiles, the whiskers extend to 1.5x the interquartile range.

**
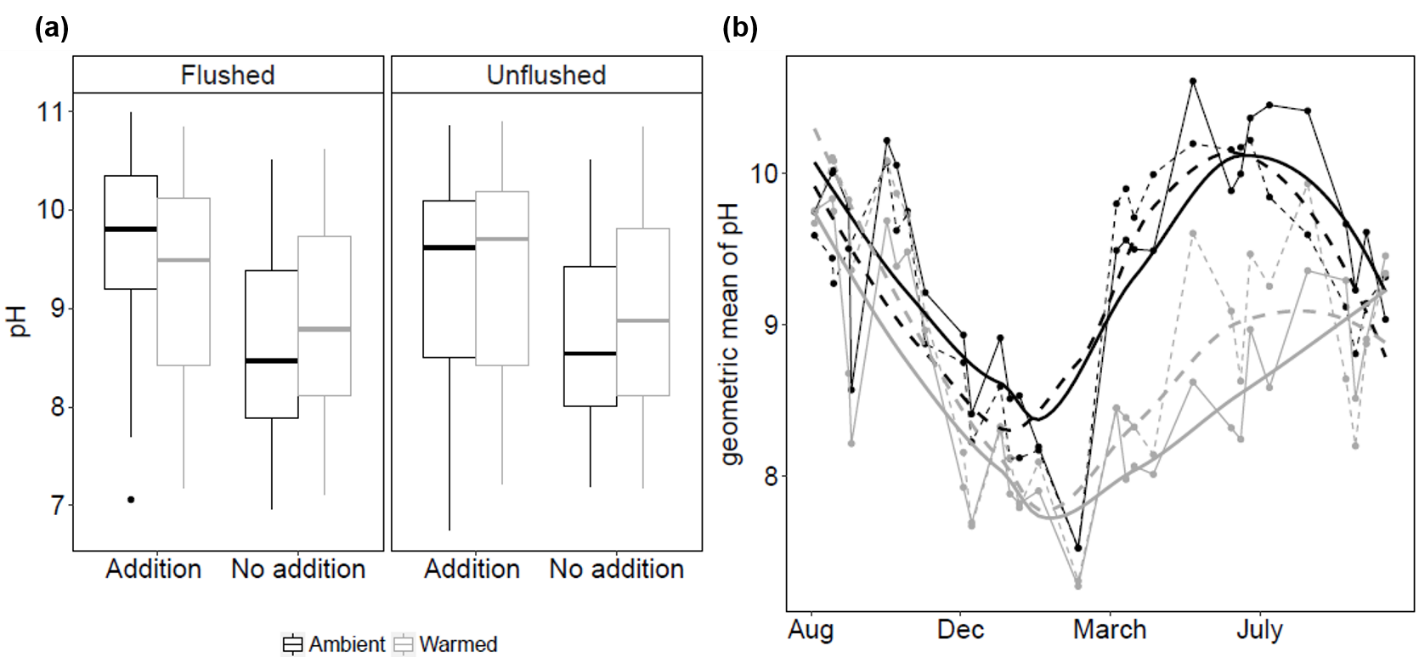
**

Fig. S10 Variation in pH among treatments. In (a) the thick line corresponds to the median, the lower and upper hinges correspond to the 25^th^ and 75^th^ percentiles, the whiskers extend to 1.5x the interquartile range. In (b) a loess smoother is shown over the geometric mean for each treatment – date combination. Black lines are nutrient enriched mesocosms (high addition), grey lines are ambient-nutrient mescososm. Solid lines are unheated mesocosms, dashed lines are heated mesocosms.

**Tables**

| Table S1. Nutrient, chlorophyll (total and cyanobacteria), pH and conductivity measurements from Blea Tarn reservoir water used for flushing. Data are not available for the final flushing event (August 2015). | | | | |
| --- | --- | --- | --- | --- |
|  | Sep-14 | Nov-14 | Feb-15 | May-15 |
| TP (mg L^-1^) | <LOD | <LOD | <LOD | 0.05 |
| TN (mg L^-1^) | 0.33 | 0.45 | 0.46 | 0.36 |
| SRP (µg L^-1^) | 0 | 11.66 | 7.60 | 3.50 |
| NO3 (mg L^-1^) | 0.22 | 0.34 | 0.35 | 0.33 |
| Chlorophyll-*a* (µg L^-1^) | 0 | 1.61 | 0.95 | 0.54 |
| pH | 7.44 | 7.84 | 7.66 | 8.38 |
| sp conductivity | 78 | 62 | 53 | 73 |
| Cyanobacteria chlorophyll-*a* (µg L^-1^) | n/a | 1.1 | 0 | 0 |

| Table S2. Three way ANOVA of between treatment differences in chlorophyll-*a* at the first time point of the experiment. | | | | | |
| --- | --- | --- | --- | --- | --- |
| Treatment | Df | Sum Sq | Mean Sq | F value | Pr(>F) |
| nutrient addition | 1 | 583 | 583.2 | 0.48 | 0.50 |
| Flushed | 1 | 4 | 4.3 | 0.00 | 0.95 |
| Warming | 1 | 2817 | 2817.5 | 2.31 | 0.14 |
| nutrient enriched x flushed | 1 | 84 | 84.1 | 0.07 | 0.80 |
| nutrient enriched x warming | 1 | 7 | 6.9 | 0.00 | 0.94 |
| flushed x warming | 1 | 1115 | 1114.6 | 0.91 | 0.35 |
| nutrient enriched x flushed x warming | 1 | 133 | 1222.1 | 0.11 | 0.74 |

| Table S3. Paired t-test. Total chlorophyll-*a* and cyanobacteria chlorophyll-*a* concentration (μgL^-1^) before and after flushing. | | | | | | |
| --- | --- | --- | --- | --- | --- | --- |
| Variable | estimate | statistic | *p-*value | parameter | conf.low | conf.high |
| Cyanobacteria chlorophyll-*a* | 23.46 | 2.97 | **0.006** | 31 | 7.36 | 39.56 |
| Chlorophyll-*a* | 142.91 | 2.67 | **0.01** | 31 | 33.93 | 251.90 |

| Table S4. Percent (%) cyanobacteria genus biovolume of total cyanobacterial biovolume composition from four sampling events (May 5^th^, June 3^rd^, July 29^th^ and August 26^th^ 2015). | | | |
| --- | --- | --- | --- |
| Order | *genus* | % biovolume | |
| Nostocales | | 68 | |
|  | *Dolichospermum* |  | *17* |
|  | *Aphanizomenon* |  | *51* |
| Oscillatoriales | | 14 | |
|  | *Oscillatoria* |  | *<0.1* |
|  | *Pseudanabaena* |  | *13* |
|  | *Limnothrix* |  | *0.4* |
| Chroococcales | | 18 | |
|  | *Aphanothece* |  | *0.6* |
|  | *Cyanodictyon* |  | *4* |
|  | *Microcystis* |  | *13* |
|  | *Unidentified* |  | *0.2* |
